# Parallels and Overlap: The Integration of Homeostatic Signals by Mesolimbic Dopamine Neurons

**DOI:** 10.3389/fpsyt.2018.00410

**Published:** 2018-09-03

**Authors:** Ted M. Hsu, James E. McCutcheon, Mitchell F. Roitman

**Affiliations:** ^1^Department of Psychology, University of Illinois at Chicago, Chicago, IL, United States; ^2^Department of Neuroscience, Psychology and Behavior, University of Leicester, Leicester, United Kingdom

**Keywords:** ventral tegmental area, nucleus accumbens, hunger, thirst, sodium appetite, reward, motivation

## Abstract

Motivated behaviors are often initiated in response to perturbations of homeostasis. Indeed, animals and humans have fundamental drives to procure (appetitive behaviors) and eventually ingest (consummatory behaviors) substances based on deficits in body fluid (e.g., thirst) and energy balance (e.g., hunger). Consumption, in turn, reinforces motivated behavior and is therefore considered rewarding. Over the years, the constructs of homeostatic (within the purview of the hypothalamus) and reward (within the purview of mesolimbic circuitry) have been used to describe need-based vs. need-free consumption. However, many experiments have demonstrated that mesolimbic circuits and “higher-order” brain regions are also profoundly influenced by changes to physiological state, which in turn generate behaviors that are poised to maintain homeostasis. Mesolimbic pathways, particularly dopamine neurons of the ventral tegmental area (VTA) and their projections to nucleus accumbens (NAc), can be robustly modulated by a variety of energy balance signals, including post-ingestive feedback relaying nutrient content and hormonal signals reflecting hunger and satiety. Moreover, physiological states can also impact VTA-NAc responses to non-nutritive rewards, such as drugs of abuse. Coupled with recent evidence showing hypothalamic structures are modulated in anticipation of replenished need, classic boundaries between circuits that convey perturbations in homeostasis and those that drive motivated behavior are being questioned. In the current review, we examine data that have revealed the importance of mesolimbic dopamine neurons and their downstream pathways as a dynamic neurobiological mechanism that provides an interface between physiological state, perturbations to homeostasis, and reward-seeking behaviors.

## Introduction

Motivated behaviors are fundamentally rooted in homeostasis. To survive, animals, including humans, have adopted behavioral strategies to efficiently procure and ingest substances based on homeostatic perturbations, particularly deficits in body fluid (e.g., thirst) and energy balance (e.g., hunger). Historically, researchers have dichotomized physiological underpinnings of ingestive behaviors into “homeostatic” and “non-homeostatic” [occurring in the absence of need and based on positive feedback (e.g., reward; hedonic)] neural processes—that is, as separate mechanisms. Indeed, as this field progressed, there have been attempts to conceptualize these two constructs as a means through which to view maladaptive motivated behaviors, particularly behaviors underlying obesity development and drug addiction. As such, reward-related neural substrates are thought to override processes that maintain homeostatic balance (1–6). However, there is ambiguity when delineating the neural substrates that regulate “homeostatic” vs. reward processes. Rather than separable concepts, there are in fact many overlapping and parallel pathways [see (7–15) for review]. Moreover, one system traditionally viewed as participating in reward-driven ingestive behavior—the mesolimbic dopamine system—now appears to be a central hub for directing behavior in responses to homeostatic challenges. This review focuses on a particular form of mesolimbic dopamine activity—the phasic activation of dopamine neurons and subsequent phasic release of dopamine in striatal terminal regions—and will consider what these signals mean for goal-directed behavior and how they may be tuned by perturbations in physiological state.

## Phasic dopamine signaling and motivated behaviors

Midbrain dopamine neurons in VTA and SNc (substantia nigra pars compacta) exhibit distinctive firing patterns involving a combination of regular, pacemaker-like firing, irregular single spikes, and occasional high-frequency trains of action potentials, known as bursts (16–20). Bursts are of particular interest as these brief neural activations lead to transient increases in dopamine concentration in terminal regions such as NAc, which via activation of D1 and D2 receptors, influence the excitability of striatal output neurons and regulate their plasticity (21, 22). Collectively, the bursts of action potentials from dopamine cell bodies and the dopamine release associated with them (23) are termed “phasic” dopamine signaling. These signals are further shaped at the cell body and terminals by dopamine D2 autoreceptor inhibition, dopamine synthesis and vesicular packaging, the ready-releasable pool of vesicles, the rate of dopamine reuptake by the dopamine transporter [for an excellent review, see (22)], and cholinergic modulation of presynaptic release (24–26). For the purposes of this review, the primary focus will be on the preponderance of data that support phasic dopamine as critical for aspects of goal-directed behavior including reinforcement, associative strength, reward prediction, incentive motivation, value and utility (27–35).

Phasic dopamine signaling has frequently been studied in the context of motivated behaviors that result from positive reinforcement. For example, dopamine is thought to reinforce learned associations between predictive stimuli and primary reward (36, 37). As motivated behaviors occur on subsecond timescale, phasic dopamine signaling has been implicated as a driving mechanism through which mesolimbic circuitry regulates reward-seeking (38–44). Within the reward prediction error framework, phasic dopamine activity at the time of the outcome (e.g., sucrose reward) is determined by an animal's expectation. Importantly, when errors in expectation occur (e.g., the outcome is better or worse than predicted), phasic dopamine signaling at the time of reward responds with a brief change in activity, specifically a burst or pause in neuronal firing or a transient increase or suppression in dopamine release at terminal regions. Accordingly, associative strength grows steeply when differences exist between predictions and outcomes (38, 43–45) and, based on these data, phasic dopamine has been commonly termed a “teaching” signal that has functional properties in mediating motivational aspects of behavior. In turn, phasic dopamine responses evoked by predictive cues act to incentivize approach and consumption (46–50).

However, the complex roles of phasic dopamine signaling extend beyond positive reinforcement. Indeed, as goal-directed behaviors for positive reinforcement are highly adaptive, forming associations between aversive stimuli and negative reinforcement is also essential for survival. Consideration of these complexities will be critical in understanding how mesolimbic dopamine signaling can regulate behaviors in response to homeostatic perturbation as well as how changes in physiological state can profoundly change neural computations within mesolimbic circuitry. In humans, expectation of pain relief (i.e., negative reinforcement) results in transient increases in NAc blood oxygen level-dependent (BOLD) activity as well as increased functional connectivity between the NAc and key mesolimbic nodes (e.g., VTA and medial prefrontal cortex) (51, 52). Similarly, in animals, mesolimbic dopamine also play a role in processing aversive stimuli. For example, pain relief in injured rats results in increased VTA dopamine activity as measured by c-Fos immunohistochemistry and detection of NAc dopamine with microdialysis (53). Moreover, in the same study, administration of analgesia in injured rats resulted in a conditioned place preference and these effects were blocked by pharmacological inhibition of the VTA. Other research has demonstrated that cues that are associated with the avoidance of punishment (e.g., foot shock) reliably increase NAc phasic dopamine release, while inescapable punishment results in a decrease in phasic dopamine release (54). Finally, aversive agents like oral quinine and systemic LiCl (lithium chloride) reduce phasic dopamine release in the NAc and the rapid encoding of these stimuli allow for plastic adaptations in subsequent behaviors (55–58).

Mesolimbic dopamine responses to aversive stimuli comprise a substantial amount of complexity and heterogeneity (56), for example, with populations of VTA dopamine neurons that are either excited or inhibited by aversive stimuli (59). Work from Ungless and colleagues using electrophysiology in anesthetized rats has identified regional variation in the VTA, with dorsally-located dopamine neurons inhibited and ventrally-located dopamine neurons excited by aversive or noxious stimuli, while a separate population of non-dopaminergic neurons are inhibited by the same aversive stimuli (in this case, tail pinch or foot shock) (60, 61). Others have demonstrated that tail pinch in anesthetized animals increases phasic dopamine release within the dorsal striatum and NAc core while alleviation of pain by removing the tail pinch increases dopamine release in the NAc shell—converging evidence for distinct neural populations that modulate positively and negatively valence states (62).

Given that feeding and drinking may be produced through negative reinforcement processes (63–67), it is key to determine whether physiological states can exert control over mesolimbic processes. Techniques such as pharmacology and human neuroimaging, while certainly valuable, lack the temporal resolution and specificity to observe the subsecond nature of motivated behaviors and their associated mechanisms within mesolimbic pathways. Similarly, the distinctions within behavioral processes (e.g., appetitive vs. consummatory behaviors) are often difficult to parse using methods that have low temporal resolution. Thus, the combination of real-time recordings (e.g., electrophysiology, fast-scan cyclic voltammetry, *in vivo* fiber photometry) along with precise control of behavioral and physiological outcomes (e.g., intraoral and intragastric delivery of stimuli) will be critical in understanding the interactions between behavior, physiological state, and mesolimbic phasic dopamine signaling.

## Physiological and neural control of homeostasis

Homeostasis is tightly regulated by a multitude of peripheral physiological processes as well as actions within the brain. These peripheral processes include feedback from various organs (e.g., stomach and intestines; kidneys and vasculature), which use both neural (e.g., vagus nerve) and hormonal routes to relay information regarding homeostatic balance to central nodes that subsequently generate the appropriate behaviors poised to maintain and reinstate homeostatic balance (e.g., eating, drinking). Here, were provide a brief overview of the central neural processes that are traditionally thought of as “homeostatic” and how the mesolimbic system has gained prominence as a neural substrate that is sensitive to homeostatic perturbation.

### Feeding and energy balance

Energy balance is generally well-maintained by a variety of peripheral signals relating to hunger and satiety. However, feeding behaviors come with their own complexities that often deviate from traditional notions of homeostatic balance. Recent work has focused intensely on investigating digressions in homeostatic energy balance in the context of the obesity epidemic and these studies have been reviewed in a number of recent manuscripts (13, 14, 68–73). There is a rich body of literature examining neural controls of energy balance from the perspective of basic homeostatic control and perturbation as well as data suggesting that so-called “homeostatic” neural substrates are capable of regulating “reward” related feeding behaviors.

In states of hunger and satiety, hormonal mechanisms and post-ingestive effects on peripheral organs that are relayed to the central nervous system are often critical for initiating and halting feeding behaviors. Indeed, many feeding-related hormones readily enter the brain to control food intake and feeding behaviors. Hypothalamic and hindbrain nuclei have been focused on as primary targets for these hormonal and neural feeding signals. These two brain regions are traditionally associated with maintaining homeostatic energy balance, and their anatomical proximity to ventricular areas with a permeable blood brain barrier allows for heightened sensitivity to circulating hormones. The pancreas-derived hormone, insulin, a critical hormone for blood glucose regulation, enters the brain to promote satiety and reduce feeding behaviors [reviewed in (74)]. The adipose-derived satiety hormone, leptin, provides a robust satiety signal to hypothalamic and hindbrain nuclei (75–78). Similarly, the gut and hindbrain derived incretin and satiety hormone, glucagon-like peptide-1 (GLP-1), utilizes central processes to reduce food intake and feeding behavior [reviewed in (79)]. Conversely, the stomach-derived hormone, ghrelin, interacts with hypothalamic circuitry to increase food intake (80).

Perhaps most interesting, however, is the ability of these feeding hormones to engage motivated behaviors via signaling to hypothalamic and hindbrain substrates. For example, insulin administration into the arcuate nucleus of the hypothalamus reduces sucrose self-administration (81) and ventricular insulin delivery blocks high-fat diet induced conditioned place preference (82). Leptin receptor signaling in the nucleus of the solitary tract (NTS) reduces food seeking and effort to work for food (83). Similarly, GLP-1R signaling in the lateral hypothalamic area (LHA) and NTS is critical for motivated responding for food and, interestingly, chronic GLP-1R knockdown in both these regions produces elevated responding for food reward (84, 85). Ghrelin also acts on hypothalamic substrates to increase food motivated behaviors via direct ghrelin receptor activation and interactions with feeding-related neuropeptides (86–88). Importantly, these data suggest that homeostatic feeding signals act within hypothalamic and hindbrain nuclei to not only regulate feeding based on metabolic deficits, but also regulate reward-seeking and goal-directed actions.

To uncover the underlying neural mechanisms that regulate these parallel homeostatic and reward-related phenomena, agouti-related peptide expressing (AgRP) neurons in the arcuate nucleus of the hypothalamus have received a considerable amount of attention, and tremendous effort has been dedicated to examine these substrates as key regulators of energy balance. Besides the classic notion that AgRP neurons convey hunger signals (89), the ability of these neurons to engage motivated behaviors is striking. Betley and colleagues performed an elegant study examining the type of motivational signal AgRP neurons relay (66). While feeding can be motivated by intrinsic rewarding properties of food (i.e., positive valence), there is also the possibility that AgRP neurons transmit negative valence signals (i.e., hunger). Indeed, consistent with production of negative affect, optogenetic activation of AgRP neurons was shown to cause avoidance of a flavored, non-nutritive gel as well as avoidance of a location paired with AgRP photostimulation. Overall, these data again reflect the integration between homeostatic perturbation and motivated behaviors where, in this case, animals seek out food in order to restore homeostatic balance, a behavior that is in part motivated by negative valence signals. Others have demonstrated that AgRP neurons are rapidly inhibited by just the sight of food independent of consumption (90). Given the role of AgRP neurons as interoceptive sensors of hunger states, these data are initially counterintuitive. However, the authors of this study propose that this anticipatory inhibition of AgRP neurons to sensory properties of food acts to slow food-seeking behaviors once food has been found and in anticipation of restoration from caloric need. Thus, in addition to integrating signals relaying homeostatic state, AgRP neuron activity integrates learned associations with respect to food-related stimuli.

Absorption of nutrients and eventual post-ingestive consequences primarily act to engage satiety mechanisms, with hormonal signals described as one of several downstream outcomes. Alternatively, mechanisms that sense nutrients can profoundly influence motivated behaviors independently of hormonal modulation. For example, flavor-nutrient experiments have been enlightening in understanding the concept of appetition, where the post-ingestive consequences of calorie intake can induce goal-directed behaviors to further consume food. Studies from Sclafani and colleagues have demonstrated that pairing intragastric infusions of carbohydrates (e.g., glucose) or fat with particular flavors can lead to preferential intake of those flavors (91–95). There is still considerable debate about whether these food-seeking behaviors are engaged by sensing of nutrients at the level of the gut (e.g., by glucose transporters) or whether the nutrients are transported in the blood to act directly on the brain (96–98). What is remarkable is that these post-ingestive processes act independently of taste to engage neural processes that guide reward-seeking behaviors (99–103). Thus, in addition to hormonal processing, there is abundant evidence that the actions of nutrients in the periphery are critical for developing relationships between physiological state and motivated behaviors.

Taken together, these data suggest that the presumed “homeostatic” processes of hunger and satiety are complex and capable of engaging behaviors that are often considered “reward-related.” Moreover, the collective evidence is consistent with the theme that there are parallel and overlapping circuitries that integrate changes in physiological state with reward-seeking behaviors.

### Body fluid homeostasis

Body fluid homeostasis is tightly regulated as sodium deficit and dehydration pose highly threatening challenges to survival. Moreover, the rewarding value of fluids like water and hypertonic saline is heavily dependent on physiological state. A key example of this is the phenomenon of sodium appetite, where motivation to obtain and consume sodium changes drastically depending on body fluid balance. Indeed, sodium deprivation produces a robust, selective, and innate appetite for sodium that is in turn reflected in goal-directed behaviors to seek out and ingest sodium [see (104) for review]. For example, animals that have never before encountered a hypertonic sodium solution express powerful and preferential intake of it immediately upon sodium depletion (105, 106). Other studies have demonstrated that sodium depleted animals will make operant responses for sodium or approach sodium-related cues (107–109). In addition to goal-directed behaviors for acquiring salt, sodium deplete animals show exclusively appetitive taste reactivity to intra-oral infusions of hypertonic sodium in comparison to sodium replete rats that exhibit a mixture of appetitive and aversive taste reactivity (110). Thus, in states of need, the appetitive value of sodium is profoundly augmented, thus providing an ideal platform to study the impact of perturbations in homeostasis on goal-directed behaviors and reward encoding.

At the level of the central nervous system, decerebrated rats fail to produce behavioral responses to sodium depletion including increased sodium intake or frequency of saline-induced appetitive taste reactivity, relative to intact animals—suggesting an important role for forebrain structures in regulating behavioral outcomes to changes in body fluid homeostasis (111). More recent studies have revealed that a subpopulation of neurons in the NTS that express 11-β-hydroxysteroid dehydrogenase type 2 (HSD2) not only powerfully drive sodium appetite in sodium-replete mice, but also project to a number of forebrain regions that control motivated behaviors (112, 113). Other research has shown that lesions to the central nucleus of the amygdala disrupt consummatory behaviors in response to sodium depletion (114, 115), while lesions to the parabrachial nucleus result in attenuated licking to changing sodium concentrations in sodium depleted rats (116, 117). In sodium-deprived states, synchrony has been shown between the lateral hypothalamus, central amygdala, and nucleus accumbens, thus providing evidence that these regions encode the appetitive properties of sodium and its associated goal-directed behaviors based on body fluid state (118). Collectively, these data emphasize the importance of forebrain neural substrates in body fluid homeostasis and provide a valuable foundation for researchers to use sodium appetite as a powerful means to measure behaviors dependent on physiological state in the context of mesolimbic dopamine signaling.

Intake of water, like sodium, is also highly dependent on an organism's current body fluid state—this is to be expected as thirst and sodium appetite are highly intertwined and act in concert to maintain body fluid homeostasis. Centrally, circumventricular organs play a critical role in detecting blood composition and osmolarity and, under appropriate conditions, generate water seeking and consumption. Indeed, stimulation of circumventricular organs (e.g., subfornical organ, SFO) results in robust water intake in water sated animals and neural activity in these regions is modulated by water intake (119, 120). Interestingly, as the neural responses to water intake occur rapidly, it has been proposed that these processes are not directly controlled by changes in blood osmolarity (i.e., a direct response to homeostatic deficit) and are in fact an anticipatory response to changes in homeostatic balance. As such, activity in SFO nitric oxide synthase (NOS) expressing neurons is increased during water restriction and then promptly decreases seconds after subsequent water consumption (121). The decrease in SFO NOS neural activity occurs well before changes in blood osmolarity suggesting that the SFO is well equipped to anticipate changes in homeostasis. Moreover, data from the same group also suggests that suppressing activity of thirst-promoting SFO neurons is negatively reinforcing and that overall the state of thirst relays a negative-valence signal that motivates an animal to drink to terminate the aversive, thirsty state (67). These findings parallel studies on AgRP neurons of the arcuate nucleus and their responses to food and food restriction [as described above, (66)]. Interestingly, effects of the thirst-promoting hormone, angiotensin II, may, in part, require learned associations between angiotensin II receptor signaling and subsequent water intake (122). These data suggest that the central control of thirst is not limited to basic homeostatic responses and involves complex interactions between physiological state and forebrain processes that allow for the approach, consumption and reinforcement of water in order to restore body fluid homeostasis.

Taken together, there is abundant evidence suggesting that changes in physiological state, particularly those that threaten survival, can profoundly influence motivated behaviors and reward-seeking. This has critical implications for understanding the neural control of these processes and provides a platform through the interaction between homeostatic perturbation and its eventual effects on the mesolimbic dopamine system can be studied.

## Modulation of mesolimbic pathways by changes in physiological state

The mesolimbic dopamine system represents a neurobiological substrate that can adapt and respond to a variety of conditions that extend well beyond stimulus-reward associations. The data described below provide support for the hypothesis that mesolimbic phasic dopamine signaling through VTA-NAc pathways is poised to respond to changes in physiological state and is a prime example of a neural substrate that integrates both homeostatic and reward processes.

### The impact of hunger on mesolimbic dopamine signaling

Hunger is undoubtedly one of the most potent drivers of goal-directed behaviors, and a vast amount of research takes advantage of hunger as a primary means to study reward-seeking and motivated behaviors. However, it should be emphasized that hunger can powerfully modulate phasic mesolimbic dopamine signaling, can alter goal-directed behaviors toward rewards other than food (e.g., drugs of abuse), and can fundamentally impact the neurophysiology of dopamine neurons. For example, in experiments from Wilson and colleagues, rats were placed in a chamber where access to a palatable liquid meal was restricted by a wire mesh screen. After 10 min, the screen was removed, and the animal was allowed to consume the meal for 20 min. Critically, this task allowed the experimenters to use microdialysis to measure NAc dopamine levels while separating anticipatory (10 min pre-meal period) and consummatory (20 min meal access) behaviors. The results of these experiments revealed that when well-trained animals were food-deprived during a test session, there was a significant increase in NAc dopamine levels during both the anticipatory and consummatory phase of this task, relative to control rats fed *ad libitum* (123). While the authors of this study claimed that NAc dopamine release is more attributable to consummatory aspects of feeding given that they observed a more robust response during the consummatory phase of their task, the temporal resolution of microdialysis fails to capture subsecond, phasic dopamine signaling in response to food cues. Regardless, this study introduced the importance of hunger states in modulating mesolimbic dopamine signaling. Indeed, food restriction can both increase dopamine neuron firing rate (19) and reduce dopamine reuptake (124) and, in addition, food-restricted rats show enhanced extracellular dopamine release in response to extended sucrose intake (125) [however, see (126)]. Further studies have shown that chronic food restriction results in enhanced burst firing of SNc dopamine neurons, augmentation of cocaine-induced burst firing and, remarkably, persistence of this increased burst firing even after animals are refed (127). Finally, phasic NAc dopamine release evoked by sugar pellets is elevated in food-restricted rats, relative to *ad libitum* fed rats, as discussed in more detail below (128). These results have critical implications for how changes in energy balance, in this case the state of hunger, impact the physiological properties of dopamine neurons. Importantly, these findings suggest that homeostatic perturbation can (1) sensitize phasic dopamine signaling to enhance reward seeking for substances critical for survival and (2) alter phasic dopamine signaling to potentially enhance maladaptive reward seeking for substances of abuse.

As described above, alterations in energy balance can potently modulate neurobiological processes within mesolimbic neural pathways. Indeed, these changes can have a robust impact not only on goal-directed behaviors for natural rewards (e.g., food), but also enhance behavioral sensitivity toward other rewarding substances, such as drugs of abuse. Moreover, these processes are a clear example of how mechanisms that are designed to respond to homeostatic perturbations can be molded into behaviors that are maladaptive and viewed as “reward-related.” In an experiment using a model of drug relapse, rats were trained to self-administer heroin via lever presses, which was followed by a 14-day abstinence period where the rats were removed from operant chambers and underwent either mild food restriction or were allowed *ad libitum* food access. During test sessions in which the levers were available but no reward was administered (i.e., extinction parameters), food restricted rats exhibited enhanced heroin-seeking behaviors, as reflected by increased lever responses, relative to food sated rats. Furthermore, these effects can be modulated by both duration of food restriction or re-feeding. As such, reduced duration of food restriction and re-feeding attenuates this enhanced heroin-seeking behavior (129). Importantly, these data suggest that depending on the physiological state of an organism, it is possible to tune the sensitivity of mesolimbic mediated reward-seeking behaviors. Indeed, using the same behavioral paradigm, D'Cunha and colleagues demonstrated that food restricted animals exhibit increased extracellular dopamine levels in the NAc shell (measured via microdialysis) in response to the heroin-associated context (i.e., self-administration operant chambers) in comparison to sated animals. Interestingly, these effects are attenuated in response to NAc shell D1 receptor blockade, suggesting a putative role of NAc shell D1 receptor signaling in modulating hunger mediated heroin-seeking behaviors (130). These observations can be seen across other drugs of abuse and by using different reward-related behavioral paradigms. Indeed, with nicotine self-administration, the highest level of self-administration behavior can be seen in animals with the greatest degree of food and weight restriction (131). Furthermore, food restricted rats show enhanced conditioned place preference (CPP) to cocaine, relative to *ad libitum* fed rats, with food restriction potentiating both the acquisition and the expression of cocaine-induced CPP (132). Thus, in support of the data described above, food restriction and energy balance have potent effects not only on the expression of reward-seeking behaviors, but also the acquisition of goal-directed behaviors for natural rewards and a variety of drugs of abuse.

### Body fluid homeostasis and mesolimbic dopamine signaling

While much work has focused on the mesolimbic system in the context of food reward [(27, 29, 133) for examples], the role of phasic dopamine signaling in body fluid homeostasis is less well understood. However, it is important to emphasize again here that the dependency of the appetitive value of sodium or water on body fluid homeostatic state allows researchers to precisely examine the interaction between perturbations in physiological state with mesolimbic dopamine signaling and goal-oriented behaviors.

Several studies have provided a glimpse into the role of dopamine signaling in controlling body fluid homeostasis and drinking behaviors (134–137). However, few have utilized real-time recording techniques to examine (1) the effects of body fluid balance on mesolimbic pathways; (2) how changes in body fluid balance influence different components of motivated behaviors (i.e., appetitive vs. consummatory); and (3) the neurobiological mechanisms that underlie state dependent mesolimbic dopamine signaling. Early lesion studies using knife cuts demonstrated that cuts medial to the striatum result in severe dopamine depletion as well as persistent eating and drinking deficits, while cuts through the ventral and posterior portions of the striatum, while still impairing animals, had less severe consequences (134). Given the clear disadvantages of knife cut lesions, it is difficult to fully attribute these behavioral effects to striatal dopamine. However, in a separate study, more selective lesions of SNc-striatal dopamine pathways using 6-hydroxydopamine attenuated drinking behaviors in response to the thirst promoting hormone, angiotensin II (135). In pharmacological studies, dopamine receptor blockade has mixed effects. As such, systemic dopamine D2 receptor blockade was shown by one group to decrease the latency to stop drinking (136), while others showed that similar antagonism produces only modest reductions in total licking during a drinking session (137). Thus, while these data suggest a putative role of the mesolimbic dopamine system in directing drinking in response to perturbations in body fluid homeostasis, basic pharmacology and lesion studies fail to capture the subsecond processes involved in phasic dopamine responses to perturbations in body fluid homeostasis.

To carefully parse the temporal, behavioral, and mesolimbic components involved in body fluid homeostasis, recent studies from our laboratory combined real-time recording of NAc dopamine release via fast-scan cyclic voltammetry with intraoral delivery of hypertonic saline during varying states of body fluid homeostasis (138). First, naive rats that had never experienced hypertonic saline were divided into 3 groups: sodium replete, deplete, or re-replete (sodium deplete, then allowed to restore sodium balance). Critically, upon intraoral delivery of hypertonic saline, only sodium deplete animals exhibited a robust, phasic increase in NAc dopamine release and this effect was absent in both replete and re-replete animals. These data are consistent with previous work demonstrating that (1) the rewarding value of sodium is highly dependent on the physiological state of the animal and (2) that animals need not have previous experience with either sodium depletion or hypertonic saline for sodium depletion to alter the value of hypertonic saline. More importantly, these data provide strong evidence that VTA-NAc phasic dopamine signaling encodes the rewarding value of sodium in a state-dependent manner. Given the importance of VTA-NAc phasic dopamine in encoding discrepancies between predicted and actual outcomes, this study next examined whether cues associated with intraoral hypertonic saline are also capable of evoking phasic dopamine responses. Interestingly, the training history of the rats was critical. Phasic dopamine responses to sodium paired cues from rats trained only under replete conditions, were absent even when rats were subsequently tested under deplete conditions. On the contrary, phasic dopamine responses from rats trained under deplete conditions were robust when also testing under deplete conditions. However, this response appeared flexible and was absent in rats trained under deplete conditions and tested under replete conditions. Thus, both innate and learned responses to sodium are intimately connected with the physiological state of the animal. Moreover, the findings implicate an important role of VTA-NAc phasic dopamine in guiding goal-directed behaviors based on perturbations in body fluid homeostasis.

## Homeostatic signals are relayed to mesolimbic pathways

Based on the work described above, changes in physiological state and homeostatic perturbation have a key role in modulating mesolimbic pathways and their relevant behavioral outputs. What remains unclear are the gating mechanisms that (1) provide information regarding physiological state to the mesolimbic system and (2) how mesolimbic pathways integrate and relay this information. Fortunately, there are many investigations that provide insight into the mechanisms linking peripheral signals (e.g., hormonal signaling; post-ingestive feedback) and mesolimbic circuitry (discussed in Section Homeostatic signals are relayed to mesolimbic pathways) as well as the transmission of homeostatic signals through central relays to mesolimbic pathways (Section Neuronal inputs to mesolimbic pathways that regulate homeostasis).

### Direct hormonal influences on the mesolimbic pathway

Receptors for a multitude of feeding related hormones are expressed throughout the brain including key nodes within the mesolimbic pathway (77, 139–143). This provides one potential and relatively straightforward mechanism through which perturbations in homeostasis might directly influence mesolimbic physiology. For example, pharmacological activation of GLP-1Rs in the VTA, NAc core, and NAc shell reduces palatable food intake and body weight (144) and affects responses to drugs of abuse (145–147). Moreover, GLP-1R action within the VTA can alter phasic dopamine signaling as, in our laboratory, we demonstrated that LiCl-induced reductions in stimulated phasic dopamine release can be attenuated by GLP-1R blockade (58) and that ventricular injections of the GLP-1R agonist, exendin-4, can reduce cocaine-induced phasic dopamine signaling in the NAc core (148). These effects appear to be due, in part, to altered excitatory drive onto dopamine cell bodies as GLP-1R activation does not alter evoked NAc phasic dopamine release as measured in *ex vivo* brain slices (149). Other satiety hormones, including amylin and leptin, which have effects on food intake and related behaviors, are also capable of modulating phasic dopamine signaling. Indeed, in addition to VTA amylin receptor activation reducing food intake and food motivated behaviors, amylin receptor signaling in the VTA also reduces NAc core phasic dopamine release (150). Moreover, NAc core D1/D2 dopamine receptor activation partially rescues the food intake suppressive effects of VTA amylin receptor activation (151). It remains possible, though, that some of the effects of amylin may be either indirect, through action in the area postrema or via the calcitonin receptor (152). VTA amylin signaling also synergistically acts with leptin receptor signaling, where combined activation of receptors for these hormones in the VTA produces weight loss and hypophagia (153). Leptin receptor signaling in the VTA, similar to the other satiety peptides described above, can independently control energy balance and food motivated behaviors. Intra-VTA administration of leptin reduces food intake, while knockdown of VTA leptin receptors results in hyperphagia and heightened sensitivity to palatable foods (154). The sustained weight loss from VTA manipulation is yet another example of overlap between the role of the VTA in homeostatic and reward-related functions.

Interestingly, leptin and insulin signaling in the VTA can also reduce excitatory synaptic transmission on to dopamine neurons, attenuate VTA dopamine concentration, and reduce food motivated behaviors (154–159). Leptin can also exert its effects on cocaine-seeking behaviors via attenuation of cocaine-induced increases in NAc dopamine levels (160) and also reduces dopamine neuron activity (154). Furthermore, *ob/ob* mice, which lack a functional leptin gene have reduced responses to the psychostimulant, amphetamine, and have reduced dopamine release in NAc (155). Overall, these data demonstrate that leptin not only has an impact on mesolimbic pathways but is also physiologically critical for the expression of goal-directed behaviors (for either nutritive or non-nutritive substances) and appropriate functioning of phasic dopamine signaling. However, in the case of insulin, there are a few inconsistencies. In response to insulin, while some have described attenuated VTA dopamine concentration and reduced excitatory synaptic transmission (156–158), others have demonstrated increases in dopamine neuron activity and striatal dopamine release (161, 162). In light of this, the net effect of insulin on phasic dopamine activity remains unclear. One intriguing proposal is that local NAc circuits have a critical role in modulating insulin-mediated phasic dopamine signaling (162). This represents a key mechanism through which VTA and NAc dopamine signaling can independently use homeostatic signals to regulate state-dependent goal-directed behaviors.

Like satiety hormones, peripheral hunger signals can also directly act within VTA-NAc dopamine systems. Ghrelin, a stomach-derived hormone that induces feeding in sated rats (and thus is considered a peripheral “hunger hormone”), not only has receptors expressed in the VTA and NAc (142), but also alters phasic dopamine signaling and food motivated behaviors. Physiologically, ghrelin action in the VTA increases dopamine neuronal firing, synaptic plasticity, and NAc dopamine turnover (163). Pharmacological manipulations have demonstrated that intra-VTA and NAc shell delivery of ghrelin can increase food intake, however, only VTA ghrelin receptor signaling is effective in increasing food motivated behaviors (i.e. operant responding for food reward) (164, 165). One possible explanation for this is divergent circuitry from the VTA to other feeding relevant brain regions (e.g., LHA, dorsal striatum). Our laboratory has explored the effects of central ghrelin signaling on phasic dopamine release in the NAc. In awake, behaving *ad libitum* fed rats, delivery and consumption of sugar pellets reliably evoked modest phasic dopamine release in the NAc core; this release was significantly greater in food-restricted rats. Importantly, the effect of food restriction was recapitulated in *ad libitum* fed rats that were given intracerebroventricular ghrelin during the recording session. Interestingly, this effect was recapitulated by delivery of ghrelin to the LH (targeting orexin positive neurons) but not the VTA directly (128)—supporting multi-synaptic processes in driving phasic dopamine signaling. Furthermore, ghrelin's ability to potentiate phasic dopamine release extends beyond primary food reward, as central administration of ghrelin can also increase NAc phasic dopamine responses to food-predictive cues (166). Thus, by integrating hormonal signals directly within VTA-NAc pathways, mesolimbic dopamine signaling can relay information relating to both hunger and satiety states to then guide goal-directed behaviors.

### Post-ingestive caloric sensing and cues that predict calories

Besides the role of hormonal signaling within mesolimbic dopamine pathways, other post-ingestive consequences of nutrient consumption can impact central neural substrates and subsequently guide goal-directed behaviors. In regards to the neural correlates that mediate caloric sensing and cues associated with calories, mesolimbic dopamine neurons again arise as potential nodes that integrate and relay post-ingestive information.

Several investigations have determined that animals can, independently of taste, use the post-ingestive consequences of nutrient consumption (e.g., carbohydrates and fat) to generate cue-reward associations and that these processes are in part modulated by mesolimbic phasic dopamine signaling (97, 99, 167–171). For example, while animals are able to generate preferences for both sucrose and non-nutritive saccharin in comparison to water, sucrose preference is substantially greater than saccharin even when matched for sweetness. This is further emphasized in operant conditioning tasks, where animals are more inclined to lever press for nutritive substances (e.g., sucrose) over non-nutritive sweeteners such saccharin or sucralose. Importantly, in *trpm5-/-* mice, which lack sweet taste transduction, these behavioral outcomes persist only with sucrose, albeit with slower temporal occurrence. These results further suggest that motivated behaviors can occur independently of hedonic value, although hedonic value certainly acts synergistically with nutritional value (169). Furthermore, using fast-scan cyclic-voltammetry, this study revealed greater phasic dopamine signals in the NAc core to delivery of sucrose pellets than to saccharin pellets, suggesting that mesolimbic phasic dopamine is capable of encoding the nutritive value of substances (169). This is further supported by studies that examined cued associations with nutritive or non-nutritive rewards. From our laboratory, in rats conditioned to associate cues with the delivery of either sucrose or saccharin pellets, we found that sucrose cues evoked greater phasic dopamine release in the NAc core, relative to saccharin cues. Importantly, this difference was greatest when sucrose and saccharin were presented on alternate days during conditioning—giving rats the opportunity to distinguish between post-ingestive consequences of each type of reward. When the nutritive value of these rewards was masked by presenting saccharin and sucrose pellets within the same session, the attenuation of phasic dopamine release to the saccharin cues, relative to sucrose cues, was reduced although, interestingly, was not abolished (170, 172). Overall, these data suggest that while the encoding of hedonic taste value plays a role in modulating phasic dopamine signaling, nutritive value of rewards also contributes strongly to these processes.

Interestingly, mesolimbic dopamine can be highly sensitive to the precise caloric content of nutrients. Fat, similar to sucrose and glucose, also elicits post-ingestive feedback mechanisms that influence goal-directed behaviors (171, 173–175). When fat is delivered intragastrically, dorsal striatal dopamine levels increase in parallel with increasing caloric density of fat infusions, and dopamine receptor blockade impairs an animal's ability to regulate caloric intake (176). These data suggest that mesolimbic dopamine signaling not only regulates caloric sensing, but also relays a signal reflecting the magnitude of caloric content.

The mechanisms regulating caloric sensing and hormonal regulation of energy balance, while intertwined, can also exhibit dissociable processes. In experiments involving intragastric infusions of glucose, disruption of glucose metabolism with intravenous 2-DG was shown to reduce striatal dopamine levels. Interestingly, this reduction was rescued with subsequent intravenous glucose administration (168). In a separate study, delivery of low concentrations of glucose into hepatic-portal vein was shown to increase spontaneous phasic dopamine release events in the NAc shell (97). Thus, in addition to peripheral hormonal signals, which relay homeostatic state and taste information encoding hedonic value, peripheral nutrient sensing and post-ingestive feedback signals are also critical mechanisms that regulate mesolimbic dopamine signaling in response to homeostatic perturbation.

The data described above have covered dopamine signaling in response to calories in both the dorsal and ventral striatum—brain regions that have been previously attributed to dissociable functions in regards to motivated behaviors (177). Indeed, recent evidence has suggested that dorsal and ventral striatal dopamine pathways are differentially modulated by caloric content and hedonic value. As such, intake of non-nutritive sucralose was shown to increase ventral striatal dopamine levels, however, increases in dopamine within the dorsal striatum only occurred when sucralose intake was paired with intragastric glucose. Moreover, when intragastric glucose infusions were paired with the taste of a bitter compound, ventral striatal dopamine was unresponsive, relative to baseline, while dorsal striatal dopamine levels were augmented (103). Taken together, these results provide evidence for separate striatal circuits that regulate hedonic value or post-ingestive reinforcement. However, questions remain regarding whether hedonic value and caloric value are processed either through distinct dorsal vs. ventral striatal pathways or via integrated pathways within these brain regions. Regional specificity of phasic dopamine signals remains a subject of intense study (178–180).

## Neuronal inputs to mesolimbic pathways that regulate homeostasis

While physiological state information can be relayed to the mesolimbic system directly via hormones, the VTA and NAc also receive extensive neuronal projections from a multitude of neural substrates that are involved in processing homeostatic information. This provides an alternative, yet complementary, mechanism through which physiological state information is integrated prior to being transmitted to the mesolimbic dopamine system.

### Hindbrain inputs

Hindbrain neural processes are capable of modulating goal-oriented behaviors and reward (83, 84, 144). Thus, it is unsurprising that homeostatic information that is relayed to hindbrain neural substrates can be transmitted to mesolimbic dopamine pathways. Indeed, the NTS has a direct projection to both VTA and NAc (181) and homeostatic feeding circuits, such as GLP-1 expressing neurons in the NTS, provide input to the VTA (144). Interestingly, NTS GLP-1R activation alters the expression of dopamine-related genes in the VTA (182). These data suggest that, besides direct homeostatic signaling to mesolimbic circuitry, these signals can be initially gated by hindbrain processes before being relayed to the mesolimbic circuits. Further support for this hypothesis can be observed in animals with lesions to the area postrema (AP) and parabrachial nucleus (PBN) - while amylin receptor activation can reduce VTA stimulated dopamine release in the NAc in control animals, these effects are abolished in animals with either AP or PBN lesions (152).

Brainstem subregions that regulate body fluid homeostasis also have projections to the mesolimbic pathway. Within the NTS, a subset of neurons expressing 11-β-hydroxysteroid dehydrogenase type 2 (HSD2) are particularly sensitive to sodium depletion and signals that relay sodium deficiency (112) and in turn project to the AP (183). While these HSD2 neurons have sparse projections to the VTA, there are monosynaptic VTA projections to the pre-locus coeruleus (pre-LC) and external lateral PBN (PBel-inner) (184). Importantly, within the pre-LC and PBel-inner, sodium sensitive neurons that express Forkhead box protein 2 (FoxP2) have direct projections to the VTA. Thus, one logical pathway through which sodium deficiency and body fluid homeostasis is relayed to mesolimbic pathways is via a polysynaptic pathway that traverses through NTS/AP HSD2 neurons → Pre-LC/PBel-inner FoxP2 neurons → VTA dopamine neuron circuitry (104).

### Midbrain inputs

Besides hindbrain projections, the VTA also receives extensive input from midbrain substrates that regulate homeostatic processes. In particular, the lateral dorsal tegmental area (LDTg) projections to the VTA (185, 186) have been identified as one pathway that regulates homeostatic functions and goal-directed behaviors. Additionally, the LDTg is critical for maintaining both burst and tonic firing of VTA dopamine neurons (187, 188). Interestingly, animals can be trained to self-administer optogenetic activation of LDTg inputs to the VTA (189), which in turn increases NAc dopamine levels (190). Additionally, excitation of cholinergic or glutamatergic LDTg input to the VTA produces conditioned place preference for opioids (191). Overall, these data provide support for LDTg to VTA pathways in modulating reward, however, whether homeostatic changes can mediate this pathway remains understudied. Nonetheless, the LDTg is anatomically poised to use homeostatic signals. The LDTg receives input from the NTS (192) and expresses receptors for hormones that regulate energy balance (140, 141). Recent data have also demonstrated that GLP-1 (193) and amylin receptor activation (193) in the LDTg reduces food intake and motivated behaviors. Collectively, midbrain LDTg input to the VTA is critical for VTA function, and this system reflects yet another parallel pathway through which homeostasis and reward interact.

Other midbrain inputs to the VTA, including the pedunculopontine tegmental nucleus (PPTg) can modulate goal-directed behaviors and putative homeostatic functions. For example, the PPTg sends cholinergic and glutamatergic input to the VTA (194–196), modulates burst firing of VTA neurons (197), and interacts with the VTA, along with other limbic structures, to regulate reinstatement of cocaine seeking (198). In the context of homeostasis, others have demonstrated that PPTg lesions block food conditioned place preference in food-sated, but not food-deprived rats (199). Moreover, melanin-concentrating hormone (MCH) and orexin producing neurons from the LHA send projections to the PPTg (200), although the role of this pathway in modulating energy balance is unknown. Thus, while the PPTg appears to have putative roles in regulating homeostatic balance and goal-directed behaviors, the precise interactions between PPTg, homeostatic perturbations, and phasic dopamine signaling remains to be determined.

### Forebrain inputs

Hypothalamic nuclei in the forebrain, as we have briefly discussed, consist of classic homeostatic neural regulators. In parallel with hindbrain and midbrain pathways, hypothalamic brain regions, in particular the lateral hypothalamic area (LHA), send direct and reciprocal projections to the VTA and NAc (3, 201–207) and provide another set of circuits through which homeostatic signals can be relayed to the mesolimbic dopamine system. Several studies have delved into the role of LHA-VTA pathways in reward related behaviors, while others have assessed the relationship between homeostatic LHA signaling and the VTA. Photostimulation of GABAergic LHA-VTA pathways results in increased NAc dopamine release and promotes approach behaviors (208). Interestingly, animals will self-administer photostimulation of LHA-VTA pathways, an effect that is mediated by neurotensin transmission (203). Polysynaptic pathways to the NAc can also modulate reward seeking. For example, LHA CART (cocaine and amphetamine regulated transcript) neurons that project to the paraventricular thalamic nucleus (PVT) promote reward behaviors, which can then be attenuated by glutamate receptor blockade in the NAc shell (209).

In the context of homeostatic regulation, there have been some efforts to delineate the relationship between hormonal signals, the LHA, and VTA dopamine signaling. The LHA contains a subpopulation of neurons that produce orexin, a neuropeptide that interacts with feeding hormones and drugs of abuse (80, 210–223), which then project to many sites throughout the brain, the VTA among them. It has been demonstrated *in vitro* that leptin administration can reduce excitatory synaptic strength between LHA orexin neurons and the VTA and that these effects can be attenuated by fasting or high-fat diet induced obesity (224). Additionally, energy balance can be regulated through LHA neurotensin neurons that also express leptin receptors. These in turn modulate local LHA orexin neurons that subsequently impact mesolimbic pathways (225). Finally, we have demonstrated in our laboratory that the hyperphagic effects of central ghrelin administration can be blunted by intra-VTA administration of orexin receptor antagonist and that ghrelin injected directly into LHA recapitulates the effect of ICV ghrelin on phasic dopamine signaling whereas, interestingly, intra-VTA ghrelin does not (128).

Neurons in the LHA are also sensitive to levels of circulating nutrients and this may be a route by which nutritional value is relayed to mesolimbic circuitry (226–229). Along these lines, MCH neurons in LHA are excited by physiological increases in extracellular glucose (228). These neurons project to VTA and optogenetic activation of their terminals biases preference for the non-nutritive sweetener, sucralose, relative to sucrose. Interestingly, co-activation of these terminals while mice are drinking water is not sufficient to shift their preference away from sucrose, indicating that taste is involved and is presumably integrated with nutritive value as relayed by MCH neuron activation. In addition, the same manipulation, activation of MCH neuron terminals in VTA, elevates dopamine levels in NAc (230). Collectively, the data described above are a prime example of how reward-seeking is modulated by changes to physiological state and, moreover, show how hypothalamic to VTA circuits can act in concert with hindbrain and midbrain VTA pathways to dynamically regulate homeostatic responses and goal-directed behaviors.

Growing evidence now supports forebrain structures involved in executive functions such as learning, memory, and decision making, as sites for homeostatic signaling (14, 231–233). Of particular interest are pathways originating from the ventral subregion of the hippocampus (vHP), which have been shown to interact with phasic dopamine signaling (234) as well as to integrate homeostatic signals (235–237). For example, ghrelin administration to the vHP increases food intake and reward seeking and increases phosphorylated tyrosine hydroxylase within the NAc (236). Further studies have demonstrated that vHP ghrelin signaling requires downstream communication to LHA orexin neurons (238), providing putative evidence for a polysynaptic pathway between the vHP, LHA, and VTA. The vHP also has the capability of bidirectionally modulating feeding behaviors, as GLP-1R signaling in the vHP potently reduces food intake and motivated behaviors (237). Additionally, these effects are mediated by vHP to medial prefrontal cortex pathways (mPFC) (239). The mPFC to NAc projections have been implicated in a variety of phasic dopamine-related functions (240–243) and mPFC dopamine signaling has been shown to have a role in modulating energy balance and feeding (242). Together, these data provide another polysynaptic pathway (e.g. VTA→mPFC→NAc) through which homeostatic perturbation might impact phasic dopamine signaling. Overall, these data emphasize the notion that pathways that regulate homeostasis and goal-directed behaviors are remarkably complex, and the degree to which information regarding physiological state is relayed to mesolimbic dopamine pathways is not limited to peripheral, hindbrain, or midbrain input.

## Conclusions and considerations

In the current review, we have emphasized the notion that there is substantial overlap between homeostatic and reward-related neural processes. More specifically, existing data support complex, dynamic, and parallel neural pathways that integrate physiological state and goal-directed behaviors. Accordingly, mesolimbic phasic dopamine signaling represents one of many central mechanisms through which these integrative processes can occur.

However, many questions remain to be addressed. First, the intricacies between phasic burst firing of VTA dopamine cell bodies and terminal dopamine release in the NAc are under evaluation. While we have described heterogeneity of VTA dopamine neurons in processing positive vs. negative valence, the electrophysiological activity of individual NAc neurons is also intimately tuned to either rewarding or aversive stimuli and can be shaped toward cues that predict these stimuli (244). Of course, while VTA dopamine neuron activity and phasic burst firing of VTA neurons robustly mediates terminal dopamine release and reuptake (22, 245), several investigations have proposed the notion that NAc neurons are capable of modulating terminal release of dopamine independently of VTA cell bodies (24–26). For example, optogenetic activation of NAc cholinergic interneurons increases extracellular dopamine (25, 26) that is in turn modulated by the endocannabinoid system and prefrontal cortical afferents to the NAc (245). In the context of homeostatic modulation of phasic dopamine signaling, we have briefly described the effects of insulin on NAc dopamine release. Data from Stouffer and colleagues have emphasized the role of cholinergic interneurons (which express insulin receptors) in modulating the insulin-mediated increases in dopamine levels within the striatum (162). Collectively, the possibility of local NAc circuitry and NAc input from other brain regions in modulating dopamine release should be a focal topic in conjunction with perturbations in homeostasis.

Next, we have described the ability of phasic dopamine signaling to respond to a variety of different perturbations to homeostasis, however, whether the responses of the mesolimbic dopamine system to varying physiological states utilizes overlapping or distinct output pathways is unknown. Given the variety of inputs to the VTA, as we have described above, it seems highly likely that these inputs are capable of engaging distinct subpopulations of VTA neurons whose signals are subsequently integrated to generate a specific behavioral outcome. For example, in the case of feeding and energy balance, it would be enlightening to determine whether the receptors for feeding hormones are co-expressed on the same neuronal populations within the VTA and how anorexigenic and orexigenic peptides interact through local VTA circuits to impact phasic dopamine signaling. In a similar vein, the degree to which perturbations in body fluid homeostasis can impact mechanisms regulating energy balance at the level of the VTA should also be examined. Eating and drinking are intimately linked and it is well known that eating stimulates thirst and dehydration induces anorexia [excellently reviewed in (246)]. Moreover, temporal differences in signaling pathways between this mixture of hormones might also affect these interactions. Indeed, what is left to be reconciled is the slow temporal action of peripheral hormone or nutrient signaling to the brain, relative to the rapid subsecond actions of phasic dopamine signaling.

In light of the data presented here, the endogenous relevance of phasic dopamine signaling in regulating behavioral responses to homeostatic perturbations requires further study. We briefly describe work that attributes phasic dopamine signaling as a “teaching signal” that strengthens associations and guides behaviors toward stimuli that are advantageous for an animal. Indeed, some have argued that phasic dopamine signaling is in large part mediating approach/appetitive behaviors toward cues associated with high subjective utility (e.g., incentive salience) as opposed to consummatory behaviors (47, 50). The question that remains, however, is how the robust influence of homeostatic perturbation on phasic dopamine signaling subsequently impacts particular behavioral components that are related to motivation and goal direction. One possibility is that homeostatic need states tune phasic dopamine signaling to engage appetitive behaviors toward stimuli that are relevant for the need state. For example, in a sodium deplete state, this physiological state might tune phasic dopamine signaling to engage appetitive behaviors for obtaining sodium, while responses for food reward are attenuated. Whether VTA-NAc pathways are physiologically relevant for these behavioral outputs remains to be seen. Thus, for future studies, researchers might use loss-of-function experiments (e.g., optogenetic inhibition of dopamine neurons) to determine what characteristics of goal-directed behaviors (e.g., appetitive, consummatory) are impacted during varying states of need.

Finally, the interaction between sex differences, homeostasis, and phasic dopamine signaling requires extensive examination. Several recent studies have demonstrated that the effects of central homeostatic signaling are sex dimorphic. For example, female rats have higher levels of LHA ghrelin receptor expression than males, and acute blockade of LHA ghrelin receptors in females, but not males, reduces food intake, body weight, and food seeking behaviors (88). Central GLP-1R activity also reveals sex dimorphism, where broad activation of GLP-1Rs results in greater suppression in food motivated behaviors in female compared to male rats along with interactions with estrogen signaling (247). Interestingly, these results appear to vary depending on brain region, as LHA GLP-1R knockdown or blockade increases food motivation only in male rats (85). Examination of sex differences in body fluid homeostasis are also in progress. Recent studies have revealed an effect of sex on thirst, including increased water intake in male rats compared to female rats in response to angiotensin II (248), a lack of desensitization to repeated angiotensin II administration in female rats (249), and interactions of thirst and estrogens (250). Thus, future work should examine whether these sex dimorphisms in homeostatic regulation are reflected in mesolimbic phasic dopamine signaling.

While questions remain, a putative mechanism arises whereby neurons in the VTA are readily able to burst fire in response to homeostatic perturbation and the presence of state-relevant stimuli (e.g., food, cues predicting food); this in turn modulates the degree to which phasic dopamine increases occur in striatal targets—in particular the NAc. The result of the phasic increase could be to alter ongoing NAc activity as well as plasticity in the service of guiding motivated behaviors. Future research conducted with a special emphasis on the impact of physiological state on mesolimbic dopamine signaling will be critical in furthering our understanding of maladaptive behaviors with the eventual goal of effectively treating prominent health issues such as obesity and drug addiction.

## Author contributions

TH, JM, and MR conceived the scope of the manuscript. TH generated drafts. JM and MR provided critical discussion, edits and comments. TH, JM, and MR approved the final version of the manuscript.

### Conflict of interest statement

The authors declare that the research was conducted in the absence of any commercial or financial relationships that could be construed as a potential conflict of interest.
